# The loss of cardiac SIRT3 decreases metabolic flexibility and proteostasis in an age-dependent manner

**DOI:** 10.1007/s11357-022-00695-0

**Published:** 2022-12-03

**Authors:** Ping Li, Maria F. Newhardt, Satoshi Matsuzaki, Craig Eyster, Atul Pranay, Frederick F. Peelor, Albert Batushansky, Caroline Kinter, Kumar Subramani, Sandeep Subrahmanian, Jasimuddin Ahamed, Pengchun Yu, Michael Kinter, Benjamin F. Miller, Kenneth M. Humphries

**Affiliations:** 1 Aging and Metabolism Research Program, Oklahoma Medical Research Foundation, 825 NE 13thSt, Oklahoma City, OK 73104 USA; 2 Cardiovascular Biology Research Program, Oklahoma Medical Research Foundation, Oklahoma City, OK USA; 3 Department of Cardiology, Central South University, The Third Xiangya Hospital, Changsha, Hunan China; 4 Department of Biochemistry and Molecular Biology, University of Oklahoma Health Sciences Center, Oklahoma City, OK USA; 5 Ilse Katz Institute for Nanoscale Science & Technology, Ben-Gurion University of the Negev, Beer Sheva, Israel

**Keywords:** Flexibility, Proteostasis, Mitochondria

## Abstract

SIRT3 is a longevity factor that acts as the primary deacetylase in mitochondria. Although ubiquitously expressed, previous global SIRT3 knockout studies have shown primarily a cardiac-specific phenotype. Here, we sought to determine how specifically knocking out SIRT3 in cardiomyocytes (SIRTcKO mice) temporally affects cardiac function and metabolism. Mice displayed an age-dependent increase in cardiac pathology, with 10-month-old mice exhibiting significant loss of systolic function, hypertrophy, and fibrosis. While mitochondrial function was maintained at 10 months, proteomics and metabolic phenotyping indicated SIRT3 hearts had increased reliance on glucose as an energy substrate. Additionally, there was a significant increase in branched-chain amino acids in SIRT3cKO hearts without concurrent increases in mTOR activity. Heavy water labeling experiments demonstrated that, by 3 months of age, there was an increase in protein synthesis that promoted hypertrophic growth with a potential loss of proteostasis in SIRT3cKO hearts. Cumulatively, these data show that the cardiomyocyte-specific loss of SIRT3 results in severe pathology with an accelerated aging phenotype.

**Supplementary Information:**

The online version contains supplementary material available at 10.1007/s11357-022-00695-0.

## Introduction

The sirtuins are a family of NAD^+^-dependent deacylases that are implicated in lifespan extension [1]. Despite extensive investigation of this family of enzymes (reviewed in [2]), the role of SIRT3 in mammalian longevity remains incompletely understood. SIRT3 is localized to the mitochondrial matrix and its activity is the primary means of reversing protein acetylation. This is important because mitochondrial proteins are highly susceptible to non-enzymatic acetylation via the reaction of acetyl-CoA with lysine [3]. Because this reaction is so pervasive, it is proposed that a primary role of SIRT3 is to mitigate excessive acetylation, viewed as “carbon stress” analogous to how antioxidant enzymes counter oxidative stress [4, 5]. Cardiac levels of SIRT3 decline with age in mouse [6, 7] and in humans age and a sedentary lifestyle are associated with loss of SIRT3 [8]. Other reports have shown that heart acetylation is significantly elevated with age [9, 10]. This likely has contributions from the age-dependent loss of NAD^+^, a required SIRT3 substrate [2, 10–12]. 

Numerous substrates of SIRT3 have been identified and many of them are enzymes involved in metabolism, redox biology, and stress responses. Depending upon the site of lysine modification within the enzyme, acetylation can have either inhibitory or activating effects. The importance of SIRT3 in maintaining cardiac health is demonstrated in animal models. Whole-body SIRT3 knockout mice develop age-related pathologies in the heart, including hypertrophy and fibrosis [13], accentuated cardiac damage following ischemia and reperfusion [9, 14], and an overall reduction in life expectancy [15].

Cardiac metabolism changes in the heart with age and in general, there is more reliance on glycolysis and less on oxidative phosphorylation for ATP production [16, 17]. SIRT3 and metabolism are inextricably linked, and thus, the age-dependent loss of SIRT3 activity may contribute to metabolic changes. In support of this notion, SIRT3 global knockout mice have decreased cardiac respiratory function [14]. However, it is unknown whether the loss of SIRT3 contributes to age-dependent decreases in cardiac metabolic flexibility in which the heart begins to rely more on glucose as an energy source [18].

In addition to changes in metabolism, aging is also associated with loss of proteostasis. Proteostasis refers to the dynamic processes contributing to proteome fidelity [19]. It is comprised of a highly coordinated network that regulates protein synthesis, folding, and degradation [19]. These processes may differ depending upon the subcellular location. For example, mitochondria containing damaged protein may be turned over in bulk by the process of mitophagy [20]. Mitochondria also have localized machinery, such as LON proteases [21], that degrades oxidized and damaged proteins. Recent work has shown that, like the cytoplasm, mitochondria also contain ubiquitinylated proteins and that ubiquitin-conjugating enzymes are found in the mitochondrial matrix [22]. Although it is unknown how the loss of cardiac SIRT3 affects cardiac proteostasis, SIRT3 has a known role in regulating mitophagy [23].

In the present study, we used a mouse model with cardiomyocyte-specific deletion of SIRT3 to test the hypothesis that the loss of SIRT3 accelerates cardiac aging by decreasing mitochondrial metabolic flexibility and proteostasis. We report that cardiomyocyte-specific deletion of SIRT3 results in a severe cardiac aging phenotype by 10 months, which includes decreased cardiac systolic function and hypertrophy. Biochemically, hearts have a distinct metabolic phenotype characterized by increased reliance on pyruvate as an energy source. Using an in vivo labeling technique, we show there is also a temporal decrease in proteostasis.

## Methods

### Cardiomyocyte-specific SIRT3 knockout mice

All animal experiments were approved by the OMRF Institutional Animal Care and Use Committee. Mice were maintained on chow diet with a 12-h light/dark cycle. To generate cardiomyocyte-specific SIRT3 knockout mice, SIRT3 flox/flox mice (gift of Dr. Eric Verdin, Buck Institute; [24]) were bred with αMHC-Cre mice on a C57BL/6 J background (Jackson Laboratory, #011,038; [25]). The effects of SIRT3 knockout were similar between sexes and therefore both male and female mice are included in all data. Mice lacking SIRT3 lived to approximately 13 months of age. Mice were euthanized by overdose isoflurane or cervical dislocation.

### Cardiomyocyte isolation

Cardiomyocytes from adult mice were isolated and cultured as previously described [26, 27]. In brief, mice were euthanized by overdose of isoflurane, after which the heart was excised, the aorta was cannulated, and the heart perfused with type II collagenase (Worthington Biochemical, LS004176). Hearts were then removed and teased apart into single-cell suspension. Calcium was reintroduced gradually before cells were seeded on laminin-coated plates. Culture media was switched to a serum-free culture medium (essential medium with Hanks’ balanced salt solution), supplemented with penicillin-G, glutamine, BSA, NaHCO_3_, and butanedione monoxime (Sigma, B0753). Cells were cultured at 37 °C and 5% CO_2_.

### Isolation of heart mitochondria

Mouse heart mitochondria were isolated as previously described [28]. Briefly, after euthanasia by cervical dislocation, the chest cavity was opened and the heart was perfused, via injection into the left ventricle, with ice-cold isolation buffer containing 210 mM mannitol, 70 mM sucrose, 5.0 mM MOPS, and 1.0 mM EDTA (pH 7.4). The hearts were then excised and homogenized in 5-mL isolation buffer using a Potter–Elvehjem tissue grinder. The homogenate was spun at 500 × *g* for 5 min at 4 °C, and the supernatant was collected and passed through cheesecloth, then spun at 5000 × *g* for 10 min. The resulting mitochondrial pellet was resuspended with 60 µL isolation buffer. Isolation buffer and samples were kept on ice. The protein concentration of mitochondria was measured by BCA method (Thermo Scientific).

### Mitochondrial respiration assay

Freshly isolated mitochondria were diluted to 0.25 mg/mL in respiration buffer (210 mM mannitol, 70 mM sucrose, 10 mM MOPS, 5.0 mM KH_2_PO_4_, and 0.5 mg/mL BSA, pH 7.4), with either 1.0 mM malate plus 0.1 mM pyruvate or 30 µM palmitoylcarnitine plus 1.0 mM malate, or 10 mM glutamate plus 1.0 malate. Respiration was measured with a fluorescence lifetime-based dissolved oxygen system (Instech) at 20 °C. State 3 respiration was initiated by the addition of ADP (0.5 mM) at 2 min and followed until all ADP was converted to ATP (state 4).

### Western blot analysis

Proteins from heart tissue were harvested in isolation buffer and homogenized by a Potter–Elvehjem homogenizer. After centrifuging at 500 × *g* for 5 min, supernatants were added 1 × Halt protease/phosphatase inhibitor mixture (Thermo Fisher Scientific) before being stored at – 80 °C. Samples were separated on 4–12% NuPAGE Bis–Tris gel (Thermo Fisher Scientific) and transferred onto a nitrocellulose membrane. The blot was blocked with Odyssey TBS blocking buffer (LICOR). Intensities of bands were standardized to the total protein loaded (Revert Total Protein Stain; LI-COR, 926–11,014). The blot was probed with primary antibodies overnight at 4 °C. Protein signals were detected with secondary antibody (IRDye 800CW, LI-COR; 1:5000 dilution) and analyzed on an Odyssey CLx imaging system using Image Studio software (LI-COR). Primary antibodies against Sirt3, anti-acetyl lysine, BCKDH, phosphorylated BCKDH (Ser293), PDH, pPDH (Ser293), AKT, pAKT (Ser473), mTOR, p-mTOR (S2448), p70-S6K, phosphorylated p70-S6K (Thr389), 4E-BP1, p4E-BP1 (Thr37/46), and LC3 A/B were purchased from Cell Signaling Technology.

### Pyruvate dehydrogenase (PDH) activity assay

PDH activity was measured as previously described [28, 29]. Freshly isolated mitochondria were diluted to 0.05 mg/mL with a buffer containing 0.05% Triton X-100 and 25 mM MOPS (pH = 7.4). PDH activity was determined spectrophotometrically as the rate of NAD^+^ reduction to NADH at 340 nm upon addition of 1.0 mM NAD^+^, 2.5 mM pyruvate, 0.2 mM thiamine pyrophosphate, 0.1 mM CoASH, and 5.0 mM MgCl_2_ (pH = 7.4).

### Cardiac histology

Heart tissues were fixed in 10% neutral buffered formalin (24 h)and embedded in paraffin. Five-micrometer sections were prepared and stained with hematoxylin and eosin or Masson’s trichrome. Images were acquired using Zeiss inverted microscope and Image J was used for analysis.

### Metabolic profiling by GC–MS

The blood was collected by cardiac puncture. Heart tissue and serum were snap-frozen with liquid nitrogen and stored at – 80 °C. Heart tissues were pulverized using grinder with metal beads. Metabolites were extracted, derivatized, and the analysis was performed as previously published using GC–MS (Agilent 7890B-5977A) [30]. Ribitol was used as an internal standard. For analysis, the serum raw data were normalized by ribitol. Heart raw data were normalized by tissue weight and the median of the total ion chromatogram. All raw GC–MS data is available upon request.

### Proteomic analysis

Samples were digested in gel using standard methods previously described [29]. Briefly, 20 μg of each sample were immobilized in a short run 12.5% SDS-PAGE gel (Criterion, Bio-rad) then fixed and stained with Coomassie blue (Pierce). Each gel lane was cut out as a single sample (∼ 1 mm^3^ pieces) and made into smaller pieces which were then washed and de-stained. Next, proteins were reduced, alkylated, and digested with trypsin. Following peptide extraction with 70% methanol/5% acetic acid in water, the extracts were dried and reconstituted in 1% acetic acid. Samples were then analyzed using the TSQ Quantiva system (Thermo Scientific). The HPLC was an Ultimate 3000 nanoflow system with a 10 cm × 75 μm i.d. C18 reversed phase capillary column that was packed in our laboratory in a New Objective Picofrit tip with a 10-μm tip opening. The packing material was Phenomenex Aeris 3.6 μm Peptide XB-C18 100A. Aliquots of 5 μL were injected and the loading phase transferred the sample from the injection loop to the column at 1.25 μL/min for 10 min. The peptides were eluted with a 60-min gradient of acetonitrile in 0.1% formic acid. Data were acquired in the selected reaction monitoring (SRM) mode for our standard targeted quantitative proteomics panels [31–33]. Additionally, data was also acquired using high-resolution accurate mass (HRAM) for potential re-interrogation in the future as needed. Data were analyzed using established Skyline methods [34]. Protein abundance was determined by normalization to BSA, a nonendogenous internal standard. Hspd1, Mdh1, and Gpi1 were used as housekeeping proteins for normalization. Methods for proteomics assay development and data processing were performed as previously reported [29]. For data and pathway analyses, proteins that had statistically significant differences in protein abundance between experimental groups were first identified. These proteins were then subjected to KEGG pathway analysis. All raw proteomics data are available upon request.

### Echocardiography

Systolic heart function indices (interventricular septal wall thickness (IVS), LV internal dimensions (LVID), and posterior wall thickness) were measured by echocardiography using Vevo 2100. Fractional shortening and ejection fractions were calculated as previously described [35, 36].

### Glycolysis assay

Glycolysis rates were measured as described in [37]. Briefly, adult cardiomyocytes cultured in 12-well plates were incubated with 1 mL/well freshly prepared culture medium containing 80 uCi/mmol [5-3H] glucose (Perkin Elmer) with or without 5 µg/mL insulin (Human, Sigma 19,278) for 2 ~ 3 h. Then, 0.8 mL/well was transferred into glass vials with hanging holders and filter papers soaked with water. After incubation at 37 °C and 5% CO_2_ for 3 days to reach saturation, filter papers were removed and placed into scintillation cocktail to detect the amount of evaporated ^3^H_2_O.

### Protein and DNA turnover determination

Mice were administered deuterium oxide (D_2_O), as previously described, to determine the synthesis rates of cytoplasmic and mitochondrial protein and DNA [38]. Briefly, 3-month and 10-month-old mice received an intraperitoneal bolus injection of 99% isotonic D_2_O (Sigma-Aldrich) calculated to enrich the body water pool to approximately 8%. Mice were then allowed ad libitum water containing 8% D_2_O for the next 13 days. At this endpoint, mice were euthanized by isoflurane overdose and blood and heart tissues were harvested rapidly and snap-frozen in liquid nitrogen for later analyses.

Heart tissue was fractionated to cytoplasmic and mitochondrial proteins by using differential centrifugation as previously described [39, 40]. The pentafluorobenzyl-N,Ndi (pentafluorobenzyl) derivative of alanine was analyzed on an Agilent 7890A GC (Agilent, Santa Clara, USA) coupled to an Agilent 5975C MS (Agilent, Santa Clara, USA) as previously described [39, 40]. To assay body water enrichment, serum was analyzed on LWIA-45-EP (Los Gatos Research).

Determination of DNA turnover was performed as described previously [38]. DNA was extracted from about 20 mg heart tissue or bone marrow suspension (DNA Mini Kit, Qiagen). DNA was hydrolyzed at 37 °C overnight and then acetylated with acetic anhydride and methylimidazole. Dichloromethane extracts were dried, then suspended with ethyl acetate and analyzed by GC/MS (Agilent 7890A GC, Agilent 5975C MS).

The protein/DNA synthesis ratio was used as an indicator of proteostatic mechanisms [38, 41]. It represents the contribution of protein synthesis for proliferation (growth) versus maintaining existing structures.

### Statistics

Data are presented as mean ± SEM. An unpaired or paired t test was used to compare two groups. When more than two groups were compared, two-way ANOVA followed by Tukey’s multiple comparisons test was performed. *p* < 0.05 was considered statistically significant. Data is available upon request.

## Results

### Cardiomyocyte-specific deletion of SIRT3 results in age-dependent hyperacetylation and cardiac hypertrophy

**Fig. 1:**
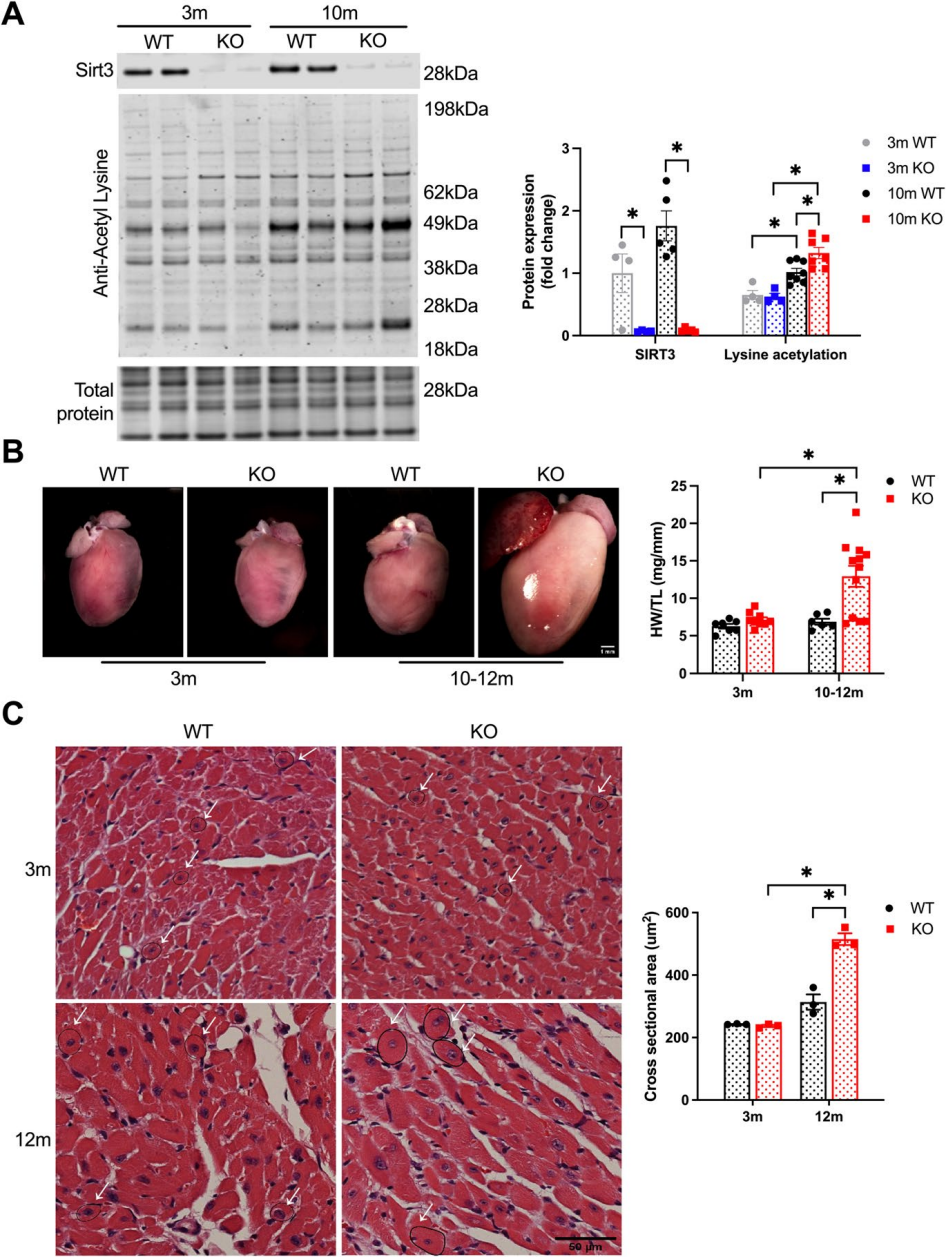
Cardiac-specific SIRT3 deletion induces cardiac hypertrophy. **A** Representative Western blots (*left*) and quantitation (*right*) of Sirt3 and acetyl lysine level in total heart homogenates from WT and SIRT3cKO mice (*n* = 4–8). **B** Representative gross image of hearts (*left*) (bar = 1 mm) and quantification (*right*) of heart weight (HW) normalized to tibia length (TL) in young 3 months old and older (10–12 months old) WT and SIRT3cKO mice (*n* = 6–12). **C** H&E-stained representative cross-section images (*left*) and quantification (*right*) of hearts in WT and SIRT3cKO mice at 3 and 12 months. Arrows indicate typical fibers with central and round nuclei for comparisons (bar = 50 µm). Fibers with central and round nuclei were used for measuring cross-sectional areas. *n* = 3 per group, 15–25 fibers per heart

Cardiomyocyte-specific SIRT3 knockout mice were generated by crossing homozygous floxed SIRT3 mice (SIRT3^fl/fl^) with Myh6-Cre-expressing SIRT3^fl/fl^ mice. Litters were comprised of a mix of SIRT3^fl/fl^ Cre^−^ and Cre^+^ mice (referred to as WT and SIRT3cKO hereafter), born at expected Mendelian ratios. Cre-expressing mice had no detectable SIRT3 as compared to SIRT3^fl/fl^ littermate controls (Fig. 1A). At 3 months, SIRT3cKO hearts had similar levels of lysine acetylation as control mice. However, at 10 months, lysine acetylation levels were significantly elevated in SIRT3cKO relative to WT. Interestingly, both WT and SIRT3cKO mice had significantly more acetylation at 10 months than at 3 months.

The absence of SIRT3 resulted in age-dependent cardiac pathology. As shown in Fig. 1B, 3-month mice had similar heart weights between groups, but older mice exhibited significant hypertrophy. At 10–12 months of age, SIRT3cKO heart weights were more than double their littermate controls. Additionally, histological analyses revealed a significant increase in cross-sectional area of cardiomyocytes in older, but not younger, SIRT3cKO hearts (Fig. 1C).

**Fig. 2:**
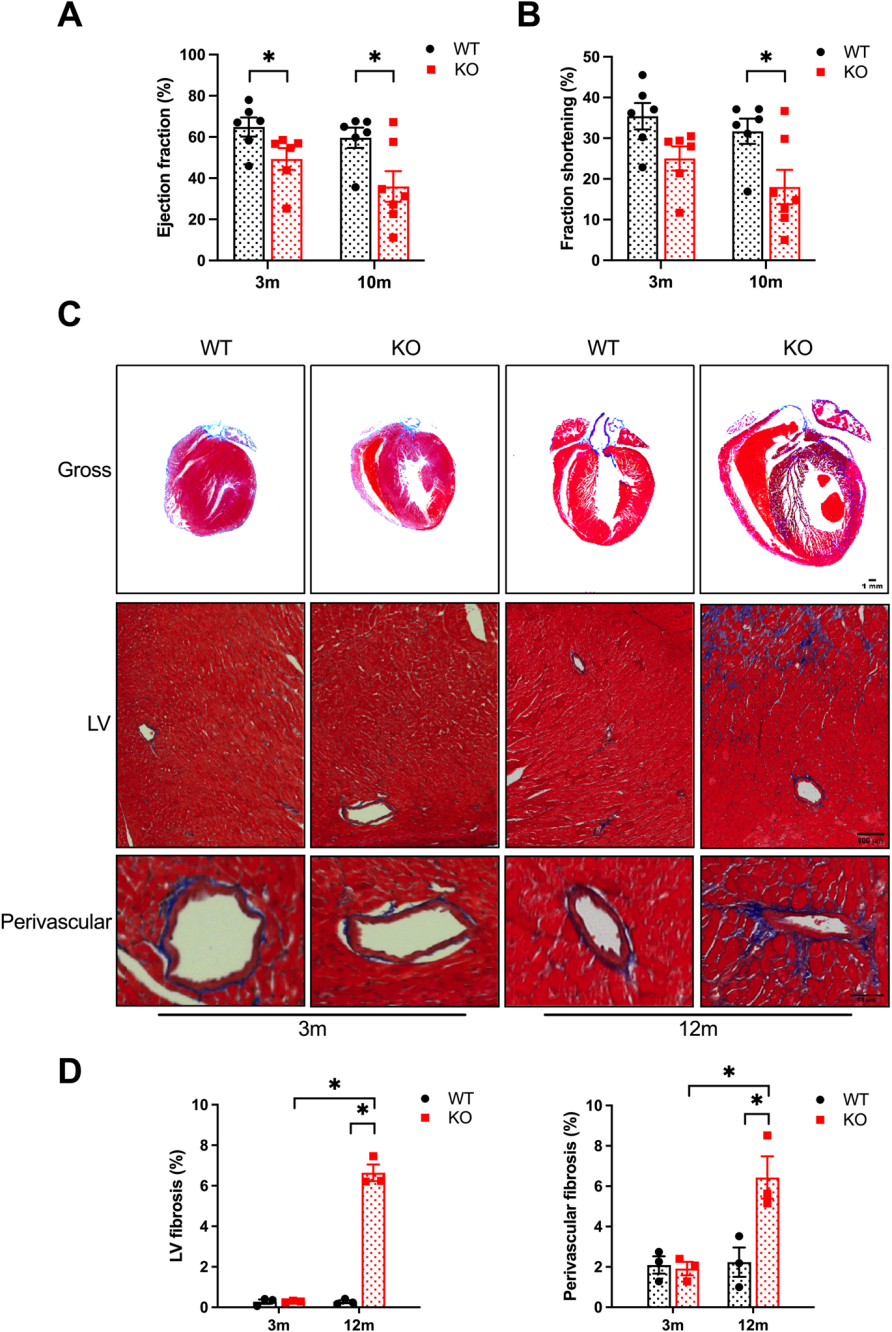
SIRT3 deletion promotes cardiac fibrosis and cardiac dysfunction. **A**–**B** Echocardiography data depicting ejection fraction (**A**) and fractional shortening (**B**) in WT and SIRT3cKO mice at 3 and 10 months (*n* = 6–7 per group). **C** Whole heart long-axis images with Masson’s trichrome staining (bar = 1 mm) and representative Masson’s trichrome. **D** Quantification of left ventricular (LV) (bar = 100 um) and perivascular (bar = 50 µm) fibrosis area in heart sections from WT and SIRT3cKO mice at 3 and 12 months. *n* = 3 per group, at least 4 images per heart. Data are presented as mean ± SEM and analyzed using 2-way ANOVA followed by Tukey’s post hoc test. *p < 0.05

An age-dependent decrease in cardiac function was also identified. SIRT3cKO hearts had decreased ejection fraction at both 3 and 10 months of age (Fig. 2A). Fractional shortening (Fig. 2B) was not statistically different in young SIRT3cKO mice, but this difference became significant at 10 months. The cardiac functional impairment was accompanied by both left ventricular and perivascular fibrosis (Fig. 2C–D) that was only apparent in older SIRT3cKO mice. Cumulatively, these results demonstrate cardiomyocyte-specific SIRT3 KO mice have age-dependent contractile dysfunction associated with hypertrophy and fibrosis.

### Mitochondrial function and substrate utilization are altered in SIRT3cKO mice

**Fig. 3:**
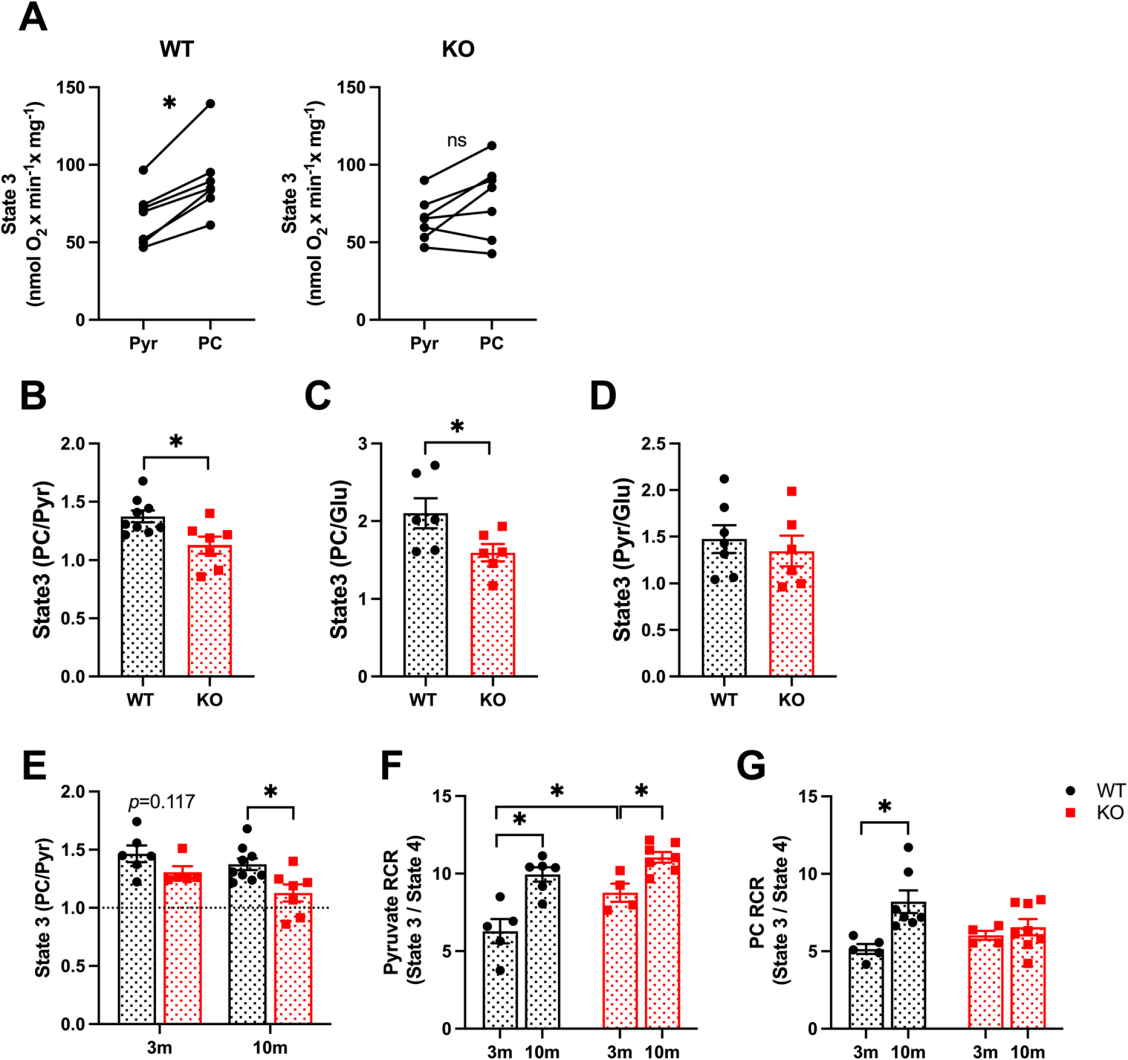
Increased pyruvate-supported mitochondrial respiration in SIRT3cKO mice. **A** State 3 respiration rate of each individual mitochondria from 10-month-old mice (*n* = 6–7 per group). **B** PC to pyruvate state3 respiration rate (PC/Pyr) from 10-month-old mice (*n* = 5–9 per group). Data from panel 3D is represented as 10 m in 3E. **C** PC to glutamate state 3 respiration rate ratio (PC/Glu) from 10-month-old mice (*n* = 6 per group). **D** Pyruvate to glutamate state 3 respiration rate ratio (Pyr/Glu) from 10-month-old mice (*n* = 6–7 per group). **E** PC to pyruvate state3 respiration rate (PC/Pyr) from WT and SIRT3cKO mice at 3 and 10 months (*n* = 5–9 per group). **F**–**G** Respiratory control ratio (RCR, calculated as State 3/State4) stimulated by pyruvate/malate (**F**) or palmitoylcarnitine/malate (**G**) with ADP (*n* = 5–9 per group). Data are presented as mean ± SEM and analyzed using paired Student’s *t* test (**A**), unpaired Student’s *t* test (**B**–**D**) and 2-way ANOVA followed by Tukey’s post hoc test (**E**–**G**). *p < 0.05. Pyr, pyruvate; PC, palmitoylcarnitine; Glu, glutamate. 3 m, 3 months old; 10 m, 10 months old

SIRT3 is a regulator of mitochondrial metabolism. Thus, we hypothesized that the age-dependent increase in cardiac pathology in SIRT3cKO mice is mediated in part by the loss of OXPHOS function and metabolic flexibility. To test this possibility, mitochondria were isolated from 10-month WT and SIRT3cKO hearts and respiratory capacity was measured with either pyruvate (Pyr) or palmitoylcarnitine (PC). In healthy hearts, the maximal rate of ADP-dependent respiration (state 3) is greater with PC than with physiological concentrations of Pyr [28, 29] corresponding to the relative preference of the heart for generating ATP via fatty acid oxidation (FAO) (Fig. 3A). This data is quantified as a ratio of PC to Pyr state 3 respiration in Fig. 3B. As compared to WT, SIRT3cKO mitochondria had similar rates of maximal respiration with either PC or pyruvate (Fig. 3A) and their ratio was significantly less than WT (Fig. 3B, 1.37 ± 0.05 for WT and 1.13 ± 0.07 for SIRT3cKO). This supports that 10-month SIRT3cKO heart mitochondria rely less on FAO as compared to WT. An alteration in metabolic substrate preference was further supported by comparing the relative rates of PC to glutamate (Glu) respiration (Fig. 3C). Again, the ratio was significantly less in SIRT3cKO mitochondria compared to WT (2.10 ± 0.19 for WT and 1.60 ± 0.11 for SIRT3cKO) while the ratio of Pyr to Glu was similar between both groups (Fig. 3D). The change in mitochondrial metabolic flexibility in SIRT3cKO hearts is progressive over time, with 3-month mice having no significant difference in mitochondrial substrate preference. However, this difference is statistically significant at 10 months (Fig. 3E).

Mitochondrial respiratory efficiency is classically described by the respiratory control ratio (RCR), a value calculated by dividing the rate of maximal, ADP-dependent respiration (state 3), by the basal rate of respiration after all ADP is converted to ATP (state 4). Distinct age and genotype-dependent changes were evident with RCRs. Heart mitochondria from 3-month SIRT3cKO mice had significantly higher RCRs with Pyr as the substrate (Fig. 3F). This suggests that Pyr is more efficiently used in 3-month SIRT3cKO mice as compared to WT. Interestingly, the RCRs also increased with age, regardless of genotype with Pyr. In contrast, only WT mice showed an age-dependent increase in RCR with PC (Fig. 3G).

### Glucose metabolism is reprogrammed in SIRT3cKO mice

**Fig. 4:**
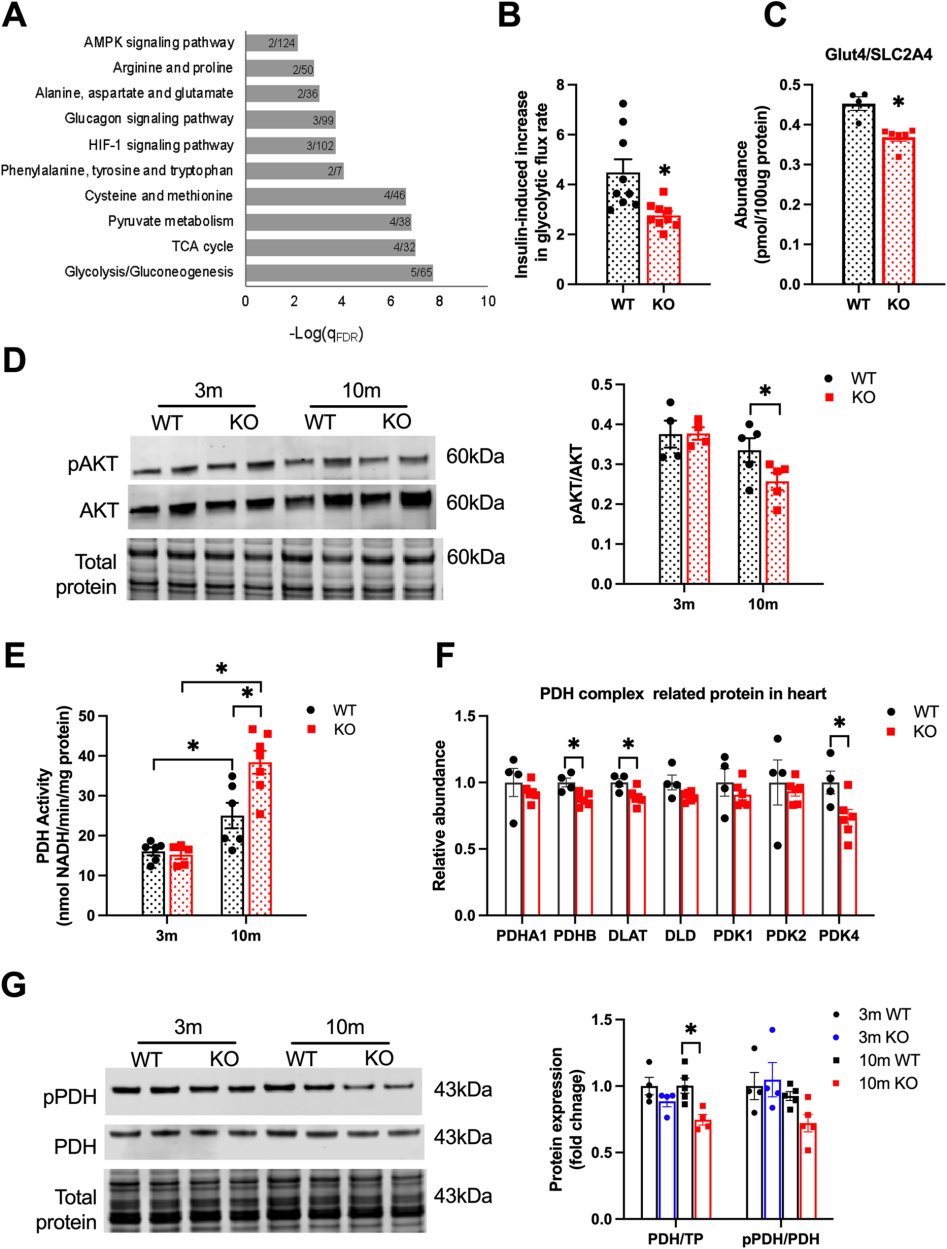
Reprogram of glucose metabolism in SIRT3cKO heart. **A** Results of the pathways analysis over KEGG database of the significantly (WT *n* = 4, KO *n* = 6) changed proteins between SIRT3cKO vs WT. Only significant pathways (qFDR < 0.05) are presented. Numbers inside the bars represent detected versus background proteins in the pathway. **B** The ratio of increasing glycolytic flux in WT and SIRT3cKO cardiomyocytes in the presence of 5ug/mL insulin (*n* = 3 per group for each experiment, 3 independent experiments). **C** Relative abundance of insulin-regulated glucose transporter 4 (Glut4) in heart (*n* = 4–6). **D** Representative western blots of phosphorylated and total Akt (*left*) and quantitation (*right*) of total Akt and phospho: total Akt ratio in hearts from WT and KO mice (*n* = 4–5). **E** PDH activity in heart mitochondria from WT and SIRT3cKO mice (*n* = 5–7 per group). **F** Relative abundance of PDH-related proteins in heart from 10-month-old mice (*n* = 4–6 per group). **G** Representative western blots of phosphorylated and total PDH (*left*) and quantitation (*right*) of total PDH and phospho:total PDH ratio in hearts from WT and SIRT3cKO mice (*n* = 4–5 per group). Data are presented as mean ± SEM and analyzed using 2-way ANOVA followed by Tukey’s post hoc test (**D**–**E**, **G**) and unpaired Student’s *t* test (**A**–**C**, **F**). *p < 0.05

To further probe the metabolic changes induced by the loss of SIRT3, targeted proteomics was conducted on 10-month-old mice. One hundred seventy proteins were quantified and then subjected to pathway analysis. As shown in Fig. 4A, proteins involved with glycolysis, TCA cycle, and pyruvate metabolism were the top pathways affected. Furthermore, significant increases in the glycolytic enzymes PFKL and ENO1 were found in SIRT3cKO hearts, while LDHB was significantly decreased (Supplemental Fig. 1 ). These changes are consistent with increased glucose metabolism in SIRT3cKO hearts that is being used to generate pyruvate.

To functionally examine if glucose metabolism is affected by the loss of SIRT3, we examined insulin-stimulated glycolysis in cardiomyocytes isolated from 10-month-old mice. Interestingly, glycolysis was more significantly enhanced by insulin in WT cardiomyocytes compared to SIRT3cKO cardiomyocytes (Fig. 4B). This was somewhat unexpected given the proteomics pathway analysis results, but it suggests SIRT3cKO mice are less responsive to further glycolytic stimulation by insulin. In support of this notion, the insulin-dependent glucose transporter, GLUT4, protein expression was decreased in 10-month-old mice SIRT3cKO (Fig. 4C). Furthermore, we observed decreased basal levels of AKT phosphorylation in older SIRT3cKO as compared to WT (Fig. 4D).

Pyruvate dehydrogenase (PDH), localized to the mitochondrial matrix, is a multienzyme complex that regulates the overall rate of glucose oxidation. We assayed PDH activity in isolated mitochondria and determined that control and SIRT3cKO mitochondria had similar enzymatic activities at 3 months (Fig. 4E). However, at 10 months, PDH activity was significantly enhanced in SIRT3cKO mitochondria (Fig. 4E). The increase in activity was not due to increased expression of the PDH enzyme subunits, as determined in our proteomics experiments (Fig. 4F). However, the expression of the PDH inhibitory kinase, pyruvate dehydrogenase kinase 4 (PDK4), was significantly lower in KO mice (Fig. 4F). Western blot was performed to examine the phosphorylation status of PDH. In comparison to the proteomics results, Western blot analysis revealed a decrease PDH (PDHA1) and showed that there was a trend towards decreased phosphorylated PDH at 10 months but failed to reach statistical significance (predetermined to be *p* < 0.05; Fig. 4G). These results indicate that the increase in PDH activity at 10-months is due, in part, to decreased PDK4 and PDH phosphorylation.

### SIRT3cKO hearts have distinct changes in branched-chain amino acids abundance

**Fig. 5:**
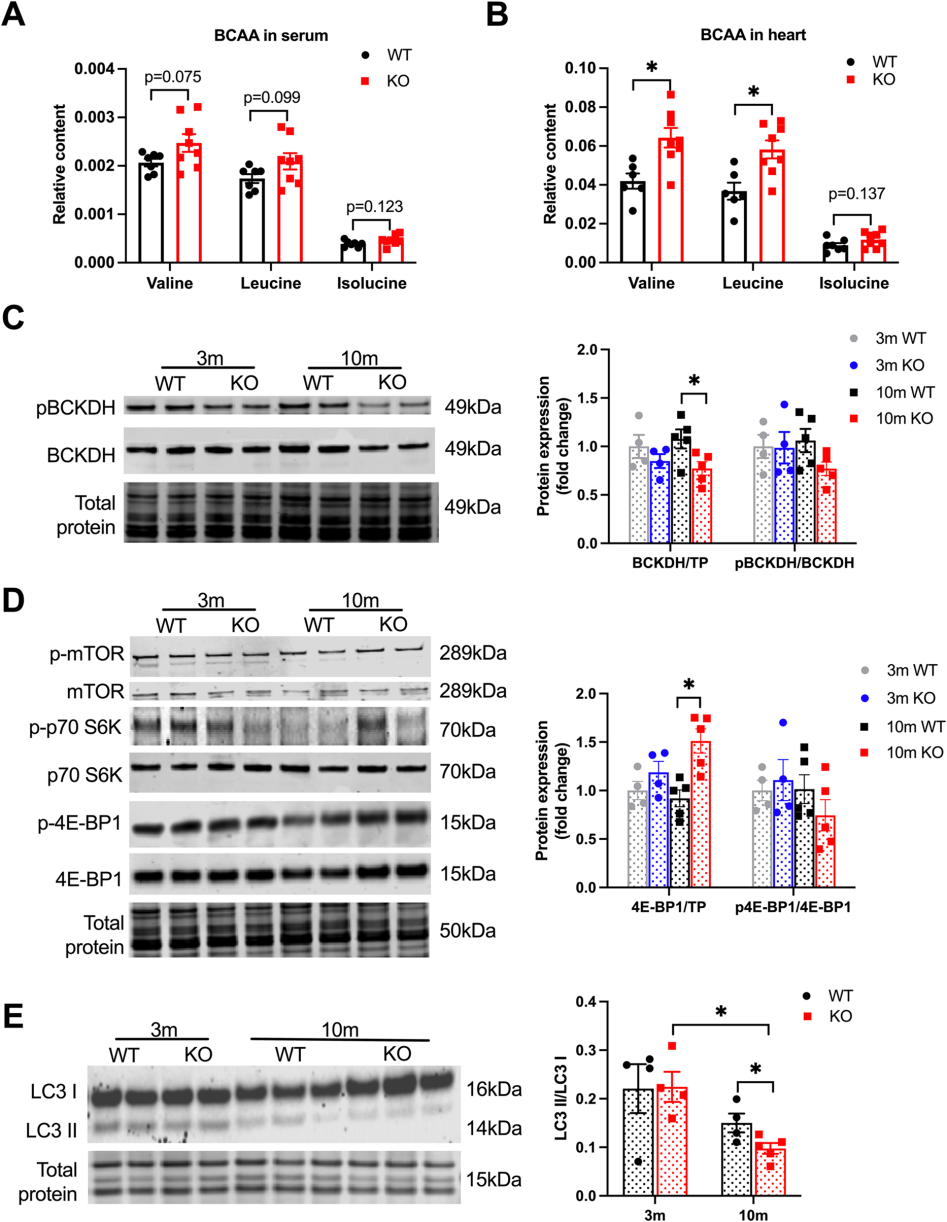
BCAA signaling is inhibited in SIRT3cKO hearts. **A-B** Content of branched-chain amino acids (valine, leucine, and isoleucine) in heart tissue and serum (n = 6–8 per group). **C** Representative Western blots of BCKDH and phosphorylated BCKDH and quantitation of total BCKDH and phospho:total BCKDH ratio in hearts from WT and SIRT3cKO mice (n = 4–5 per group). **D** Representative Western blots of mTORC1 and its downstream effector (p70S6K and 4E-BP1) and their activation states (p-p70S6K and p-4E-BP1) in hearts from WT and SIRT3cKO mice and quantitation of total and phosphorylated 4E-BP1 and their ratio (*n* = 4–5 per group). **E** Representative western blots and quantitation of LC3II and LC3I (*n* = 4–5 per group). Data are shown as mean ± SEM and analyzed using unpaired Student’s *t* test (B-C), 2-way ANOVA followed by Tukey’s post hoc test (**A**, **D**–**F**). *p < 0.05

Branched-chain amino acids (BCAAs) play an important role in the heart where they not only serve as oxidizable substrates for energy production, but they also influence the activity of key signaling pathways such as mTOR [42]. BCAAs were measured in both serum and heart homogenates by GC–MS. As shown in Fig. 5A, there was no statistically significant increase in valine, leucine, and isoleucine in the serum of SIRT3cKO mice. However, both valine and leucine were significantly higher in the SIRT3cKO heart homogenates (Fig. 5B). The enzyme complex that metabolizes BCAAs, BCKDH, is decreased in 10-month-old SIRT3cKO hearts while the inhibitory phosphorylation status was unchanged between groups (Fig. 5C). This indicates disrupted BCAA cardiac metabolism in SIRT3cKO mice.

We next examined whether the increase in BCAA is associated with an increase in mTOR signaling. We found similar levels of mTOR and phospho-mTOR in all groups (Fig. 5D and Supplemental Fig.2A The canonical mTOR substrate, p70 S6K was also similarly expressed and phosphorylated between groups (Fig. 5D and Supplemental Fig. 2B). 4E-BP1, another canonical mTOR substrate, had increased total expression in 10-month SIRT3cKO mice but was not hyper-phosphorylated (Fig. 5D). These results support that while BCAAs are elevated in SIRT3cKO hearts, there is minimal effect on mTOR signaling.

BCAAs can also impair autophagy through their activation of mTOR [43]. We therefore examined autophagy levels of hearts by measuring the ratio of LC3 lipidation (LC3-I/LC3-II) via Western blot [44]. As shown in Fig. 5E, there is an age and genotype-dependent decrease in the ratio of LC3 lipidation in SIRT3cKO mice. This finding provides evidence that there are differences in autophagy in SIRT3cKO hearts.

### In vivo* labeling to determine heart proteostasis*

**Fig. 6:**
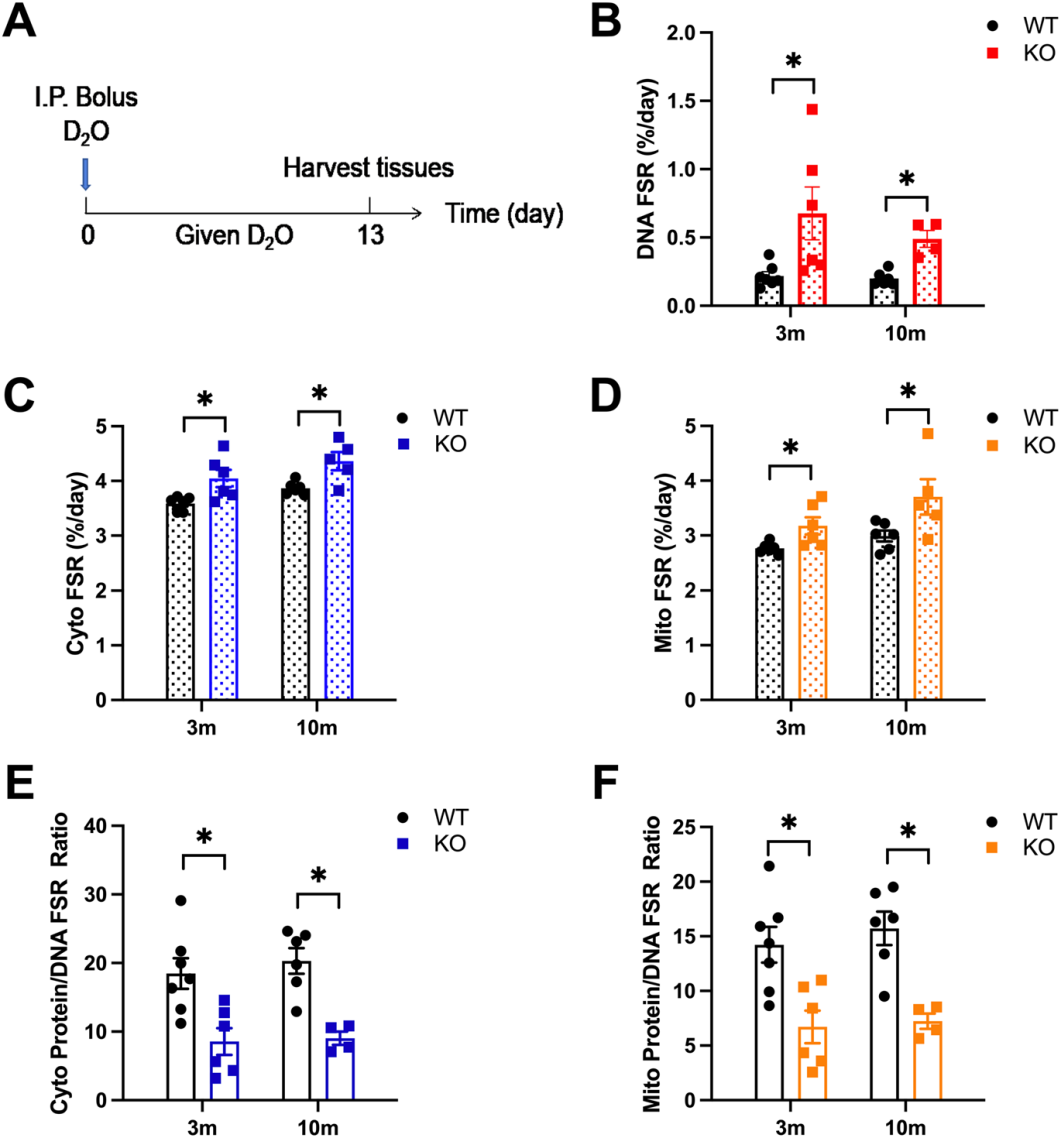
The effect of Sirt3 on protein and DNA synthesis rates. Whole heart homogenate powder was used for assaying protein and DNA synthesis rates. **A** Schematic of deuterium oxide (D2O) labeling for 13 days. **B** DNA fractional synthesis rates (FSR, %/day) in heart tissues. **C** Protein fractional synthesis rates (%/day) in cytoplasmic fractions. **D** Protein fractional synthesis rates (%/day) in mitochondrial fractions. **E** Cytoplasmic protein and DNA synthesis rate ratio. **F** Mitochondrial protein and DNA synthesis rate ratio. Data are shown as mean ± SEM and analyzed using Welch’s *t* test (**C**–**D**), 2-way ANOVA followed by Tukey’s post hoc test (**B**, **E**–**F**). *p < 0.05, *n* = 4–6 per group

In vivo deuterium oxide (D_2_O) labeling allows for the simultaneous measurement of both protein and DNA synthesis. Mice were administered D_2_O for 13 days (Fig. 6A), a time point determined from prior experiments that allows for the capture of both short-lived and intermediate-lived proteins. As shown in Fig. 6C–D, the fractional synthesis rate of proteins in both the cytoplasm and mitochondria were increased at both 3 months and 10 months in SIRT3cKO as compared to WT. However, the fractional synthesis rates of DNA were also significantly elevated in SIRT3cKO mice (Fig. 6B). The ratio of protein to DNA synthesis rates are used to determine if the measured changes in protein synthesis are impacted by cellular proliferation [45]. Indeed, these ratios were significantly decreased in both the cytoplasmic and mitochondrial fractions at both 3 months and 10 months (Fig. 6E–F). This decrease in protein to DNA synthesis ratio indicates a larger proportion of the protein synthesis was supportive of growth and that proteostasis was dramatically decreased in SIRT3cKO hearts prior to overt systolic dysfunction.

## Discussion

In the present study, we determined that there is an age-dependent loss of function, decreased metabolic flexibility, increased BCAAs, and hypertrophic growth at the cost of proteostasis in cardiomyocyte-specific SIRT3 KO mice. Global SIRT3 knockout has been described in the literature, and their phenotype includes deficits in cardiac function [23, 46]. Nevertheless, such studies cannot rule out that the global deletion of this ubiquitously expressed protein is affecting cardiac function through indirect means. Fewer studies have examined the cardiac-specific deletion of SIRT3. One study demonstrated that the ablation of SIRT3 in both skeletal and heart muscles has minimal effect on cardiac function in young mice (9-10 weeks) [47]. Another study demonstrated cardiomyocyte-specific deletion of SIRT3 increased the susceptibility of mice to HFpEF-induced diastolic dysfunction [24]. More recently and in agreement with our results here, a preliminary report supports that the cardiomyocyte-specific deletion of SIRT3 promotes heart failure [48]. To the best of our knowledge, this is the first report of the temporal changes of cardiac function, metabolism, and proteostasis in cardiomyocyte-specific SIRT3 knockout mice.

Our results show that the cardiomyocyte-specific deletion of SIRT3 is pathological, with decreased cardiac function, cardiac hypertrophy, and evidence of fibrosis. Because SIRT3 is a mitochondria-localized deacetylase, it was expected that pathology is driven in part by hyperacetylation of mitochondrial proteins. It was therefore somewhat unexpected that SIRT3cKO had only modest increases in protein acetylation. This modest increase could be due to the relative qualitative nature of western blot. Future mass spectrometry-based experiments may reveal a greater level of acetylation than observed here. Nevertheless, we do demonstrate that there is an age-dependent increase in acetylated proteins, even in WT hearts. An added nutritional stress, such as high-fat diet or diabetes, could also be examined in future studies to determine if such nutritional stresses that increase mitochondrial protein acetylation [28, 49] further exacerbate the pathology observed here in SIRT3cKO mice.

The aged heart shifts metabolism towards an increased reliance on glucose [18], and conversely, strategies that block glucose usage and restore metabolic flexibility are seen as potential longevity treatments [50, 51]. Here, we measured mitochondrial metabolic flexibility by evaluating the respiratory profile of isolated organelles with different substrates [28, 29]. We used the substrates pyruvate, PC, and glutamate, which represent primary nutrients that fuel the heart. Differences in PC-supported rates of respiration, which is mediated by fatty acid oxidation, were not apparent between WT and SIRT3cKO mitochondria. However, older SIRT3cKO mice displayed a preference for pyruvate and glutamate relative to PC (Fig. 3B–C). The preference for pyruvate was further corroborated by increased PDH activity (Fig. 4E) in SIRT3cKO hearts. Thus, cardiomyocyte-specific deletion of SIRT3 drives an aged metabolic phenotype. However, overt signs of mitochondrial dysfunction, such as reduced state 3 respiration regardless of substrate type, were not apparent. Other differences in RCRs, used to assess efficiency of mitochondria, were seen in a substrate-dependent manner between the two genotypes (Fig. 3F–G). Our respirometry results support the notion that it is critical to evaluate mitochondrial function with several different substrates to gain a more complete metabolic profile of the organelles.

PDH activity controls the overall rate of glucose oxidation. Thus, the observed increase in its activity in 10-month SIRT3cKO hearts (Fig. 4E) supports the conclusion that there is increased reliance on glucose as an energy source. The increase in PDH activity was not observed in younger hearts. It therefore cannot be concluded whether this change in metabolic profile is causative of pathological progression or if it has an adaptive mechanism in the failing heart. The increase in PDH activity is also surprising given that the enzyme levels and phosphorylation status are comparable between genotypes (Fig. 4F–G). Furthermore, increased acetylation in SIRT3cKO hearts may be expected to decrease PDH activity because of the known sensitivity of the enzyme to acetylation-mediated inhibition [52]. It is possible that the levels of hyperacetylation in SIRT3cKO hearts are below the threshold at which PDH activity is inhibited. This line of research will be the subject of future studies.

Several lines of experimentation further support that SIRT3cKO mice have differences in glucose metabolism. The experiments included direct measurement of glycolysis in isolated adult cardiomyocytes, which showed SIRT3cKO were less stimulated by insulin than WTs. Furthermore, proteomic profiling of primary metabolic pathways showed that glycolysis was the most affected pathway in 10-month-old SIRT3cKO hearts. Metabolic profiling, in contrast, revealed a striking difference between WT and SIRTcKO hearts in branched-chain amino acids. Other amino acids (Supplemental Fig. 2C) exhibited less of a genotype-specific effect, but still a trend in increased amounts in SIRT3cKO hearts that failed to reach statistical significance. Somewhat surprisingly, other intermediates in glucose metabolism and the TCA cycle were not dramatically different between WT and SIRT3cKO hearts (Supplemental Fig. 2C). Thus, the changes in amino acids, and in particular BCAAs, appear to be a specific metabolic alteration. A caveat to note, the metabolomic profiling was done by GC–MS, and a limitation of this method is that several glycolytic intermediates (sugar phosphates) are not readily detected.

The changes in branched-chain amino acids are of interest, not only because of their use as mitochondrial substrates, but also because of their bioactivity and potential involvement in longevity [53]. Specifically, there is growing interest in restricting BCAAs from the diet as a means of extending longevity [53]. Thus, the dramatic increase we observe in BCAAs in SIRT3cKO hearts could be seen as a driver of premature aging. At the cellular level, mTOR is activated by BCAAs. However, markers indicative of mTOR activity were not significantly changed in SIRT3cKO hearts while there was an indication of changes in autophagy based on LC3I-II ratios. A caveat of those measurements was that animals were not administered chloroquine prior to tissue collection, thus making it inconclusive whether autophagy was up- or downregulated, only that it differed in SIRT3cKO hearts relative to WT. However, direct in vivo measurements of protein synthesis, determined by heavy water labeling experiments, demonstrated that protein synthesis was increased in both the cytoplasm and mitochondrial fractions of SIRT3cKO hearts.

Heavy water labeling experiments are a powerful technique to determine the in vivo synthesis of protein and DNA [38, 41, 45]. We have used this approach previously to determine how aging, and potential longevity treatments (for example, caloric restriction, and rapamycin), affect proteostasis [38, 39, 41]. An important consideration when assessing age and intervention is the contributions of growth (pro-aging) versus proteostatic maintenance (slowed aging). To do this, we measure DNA synthesis as a direct measure of cellular proliferation, which is a growth-related process [38, 45]. In this study, we observed that both the cytoplasmic and mitochondrial FSRs of protein were increased in SIRT3cKO hearts (Fig. 6C–D). However, when correcting for the increased rate of DNA synthesis, we saw that the protein synthesis was more directed toward growth in SIRT3cKO with a corresponding decrease in proteostatic maintenance (Fig. 6E–F). An interpretation of this data is that at 3 months, there is already an increase in protein synthesis that promotes growth and fibrosis. Since aberrant growth often comes at the cost of protein quality control, the resultant hypertrophy does not necessarily improve function. Alternatively, the increase in DNA synthesis in 3-month mice, prior to hypertrophy and fibrosis, could also be occurring through mitochondrial-mediated endoreplication [54], a process known to drive hypertrophy. Future studies will further discern between potential mechanisms, but it is an intriguing possibility that the loss of SIRT3 drives proteostasis dysfunction, thereby causing an accelerated aging phenotype in the heart.

In conclusion, we demonstrate here that the cardiomyocyte-specific loss of SIRT3 causes the temporal progression of cardiac pathology. Mitochondria were spared from overt dysfunction, but did have changes in metabolic substrate preference, with SIRT3cKO hearts have increased reliance on glucose as an energy source. A strength and novelty of this study was applying heavy water labeling to evaluate the contributions of growth and proteostasis in SIRT3cKO mice. Significant changes in this tradeoff were obvious even in young hearts, suggesting that the loss of SIRT3 may drive an accelerated cardiac aging phenotype via changes in protein synthesis or degradation.

## Supplementary Information

Below is the link to the electronic supplementary material.

Supplementary file1 (PDF 579 KB)

## References

[1] Donmez G, Guarente L (2010). Aging and disease: connections to sirtuins. Aging Cell.

[2] Smith HJ, Sharma A, Mair WB (2020). Metabolic communication and healthy aging: where should we focus our energy?. Dev Cell.

[3] Parodi-Rullan RM, Chapa-Dubocq XR, Javadov S (2018). Acetylation of mitochondrial proteins in the heart: the role of SIRT3. Front Physiol.

[4] Wagner GR, Hirschey MD (2014). Nonenzymatic protein acylation as a carbon stress regulated by sirtuin deacylases. Mol Cell.

[5] Weinert BT, Moustafa T, Iesmantavicius V, Zechner R, Choudhary C (2015). Analysis of acetylation stoichiometry suggests that SIRT3 repairs nonenzymatic acetylation lesions. EMBO J.

[6] Li Y, Ma Y, Song L, Yu L, Zhang L, Zhang Y, Xing Y, Yin Y, Ma H (2018). SIRT3 deficiency exacerbates p53/Parkinmediated mitophagy inhibition and promotes mitochondrial dysfunction: implication for aged hearts. Int J Mol Med.

[7] Zhang J, He Z, Fedorova J, Logan C, Bates L, Davitt K, Le V, Murphy J, Li M, Wang M, Lakatta EG, Ren D, Li J (2021). Alterations in mitochondrial dynamics with age-related Sirtuin1/Sirtuin3 deficiency impair cardiomyocyte contractility. Aging Cell.

[8] Lanza IR, Short DK, Short KR, Raghavakaimal S, Basu R, Joyner MJ, McConnell JP, Nair KS (2008). Endurance exercise as a countermeasure for aging. Diabetes.

[9] Porter GA, Urciuoli WR, Brookes PS, Nadtochiy SM (2014). SIRT3 deficiency exacerbates ischemia-reperfusion injury: implication for aged hearts. Am J Physiol Heart Circ Physiol.

[10] Yeo D, Kang C, Ji LL (2020). Aging alters acetylation status in skeletal and cardiac muscles. Geroscience.

[11] Schultz MB, Sinclair DA (2016). Why NAD(+) declines during aging: it’s destroyed. Cell Metab.

[12] Walker MA, Tian R (2018). Raising NAD in heart failure: time to translate?. Circulation.

[13] Hafner AV, Dai J, Gomes AP, Xiao CY, Palmeira CM, Rosenzweig A, Sinclair DA (2010). Regulation of the mPTP by SIRT3-mediated deacetylation of CypD at lysine 166 suppresses age-related cardiac hypertrophy. Aging (Albany NY).

[14] Koentges C, Pfeil K, Schnick T, Wiese S, Dahlbock R, Cimolai MC, Meyer-Steenbuck M, Cenkerova K, Hoffmann MM, Jaeger C, Odening KE, Kammerer B, Hein L, Bode C, Bugger H (2015). SIRT3 deficiency impairs mitochondrial and contractile function in the heart. Basic Res Cardiol.

[15] Benigni A, Cassis P, Conti S, Perico L, Corna D, Cerullo D, Zentilin L, Zoja C, Perna A, Lionetti V, Giacca M, Trionfini P, Tomasoni S, Remuzzi G (2019). Sirt3 deficiency shortens life span and impairs cardiac mitochondrial function rescued by Opa1 gene transfer. Antioxid Redox Signal.

[16] Tocchi A, Quarles EK, Basisty N, Gitari L, Rabinovitch PS (2015). Mitochondrial dysfunction in cardiac aging. Biochim Biophys Acta.

[17] Lesnefsky EJ, Chen Q, Hoppel CL (2016). Mitochondrial metabolism in aging heart. Circ Res.

[18] Chiao YA, Rabinovitch PS (2015). The aging heart. Cold Spring Harb Perspect Med.

[19] Labbadia J, Morimoto RI (2015). The biology of proteostasis in aging and disease. Annu Rev Biochem.

[20] Liang WJ, Gustafsson AB (2020). The aging heart: mitophagy at the center of rejuvenation. Front Cardiovasc Med.

[21] Bota DA, Van Remmen H, Davies KJ (2002). Modulation of Lon protease activity and aconitase turnover during aging and oxidative stress. FEBS Lett.

[22] Lavie J, De Belvalet H, Sonon S, Ion AM, Dumon E, Melser S, Lacombe D, Dupuy JW, Lalou C, Benard G (2018). Ubiquitin-dependent degradation of mitochondrial proteins regulates energy metabolism. Cell Rep.

[23] Matsushima S, Sadoshima J (2015). The role of sirtuins in cardiac disease. Am J Physiol Heart Circ Physiol.

[24] Tong D, Schiattarella GG, Jiang N, Altamirano F, Szweda PA, Elnwasany A, Lee DI, Yoo H, Kass DA, Szweda LI, Lavandero S, Verdin E, Gillette TG, Hill JA (2021). NAD(+) repletion reverses heart failure with preserved ejection fraction. Circ Res.

[25] Agah R, Frenkel PA, French BA, Michael LH, Overbeek PA, Schneider MD (1997). Gene recombination in postmitotic cells. Targeted expression of Cre recombinase provokes cardiac-restricted, site-specific rearrangement in adult ventricular muscle in vivo. J Clin Invest.

[26] O’Connell TD, Rodrigo MC, Simpson PC (2007). Isolation and culture of adult mouse cardiac myocytes. Methods Mol Biol.

[27] Bockus LB, Humphries KM (2015). cAMP-dependent protein kinase (PKA) signaling is impaired in the diabetic heart. J Biol Chem.

[28] Vadvalkar SS, Matsuzaki S, Eyster CA, Giorgione JR, Bockus LB, Kinter CS, Kinter M, Humphries KM (2017). Decreased mitochondrial pyruvate transport activity in the diabetic heart: role of mitochondrial pyruvate carrier 2 (MPC2) ACETYLATION. J Biol Chem.

[29] Newhardt MF, Batushansky A, Matsuzaki S, Young ZT, West M, Chin NC, Szweda LI, Kinter M, Humphries KM (2019). Enhancing cardiac glycolysis causes an increase in PDK4 content in response to short-term high-fat diet. J Biol Chem.

[30] Batushansky A, Matsuzaki S, Newhardt MF, West MS, Griffin TM, Humphries KM (2019). GC-MS metabolic profiling reveals fructose-2,6-bisphosphate regulates branched chain amino acid metabolism in the heart during fasting. Metabolomics.

[31] Puente BN, Kimura W, Muralidhar SA, Moon J, Amatruda JF, Phelps KL, Grinsfelder D, Rothermel BA, Chen R, Garcia JA, Santos CX, Thet S, Mori E, Kinter MT, Rindler PM, Zacchigna S, Mukherjee S, Chen DJ, Mahmoud AI, Giacca M, Rabinovitch PS, Aroumougame A, Shah AM, Szweda LI, Sadek HA (2014). The oxygen-rich postnatal environment induces cardiomyocyte cell-cycle arrest through DNA damage response. Cell.

[32] Nakada Y, Canseco DC, Thet S, Abdisalaam S, Asaithamby A, Santos CX, Shah AM, Zhang H, Faber JE, Kinter MT, Szweda LI, Xing C, Hu Z, Deberardinis RJ, Schiattarella G, Hill JA, Oz O, Lu Z, Zhang CC, Kimura W, Sadek HA (2017). Hypoxia induces heart regeneration in adult mice. Nature.

[33] Rindler PM, Plafker SM, Szweda LI, Kinter M (2013). High dietary fat selectively increases catalase expression within cardiac mitochondria. J Biol Chem.

[34] MacLean B, Tomazela DM, Shulman N, Chambers M, Finney GL, Frewen B, Kern R, Tabb DL, Liebler DC, MacCoss MJ (2010). Skyline: an open source document editor for creating and analyzing targeted proteomics experiments. Bioinformatics.

[35] Meyer A, Wang W, Qu J, Croft L, Degen JL, Coller BS, Ahamed J (2012). Platelet TGF-beta1 contributions to plasma TGF-beta1, cardiac fibrosis, and systolic dysfunction in a mouse model of pressure overload. Blood.

[36] Laurence J, Elhadad S, Robison T, Terry H, Varshney R, Woolington S, Ghafoory S, Choi ME, Ahamed J (2017). HIV protease inhibitor-induced cardiac dysfunction and fibrosis is mediated by platelet-derived TGF-beta1 and can be suppressed by exogenous carbon monoxide. PLoS ONE.

[37] Yu P, Alves TC, Kibbey RG, Simons M (2018). Metabolic analysis of lymphatic endothelial cells. Methods Mol Biol.

[38] Drake JC, Peelor FF, Biela LM, Watkins MK, Miller RA, Hamilton KL, Miller BF (2013). Assessment of mitochondrial biogenesis and mTORC1 signaling during chronic rapamycin feeding in male and female mice. J Gerontol A Biol Sci Med Sci.

[39] Abbott CB, Lawrence MM, Kobak KA, Lopes EBP, Peelor FF 3rd, Donald EJ, Van Remmen H, Griffin TM, Miller BF. A novel stable isotope approach demonstrates surprising degree of age-related decline in skeletal muscle collagen proteostasis. Function (Oxf). 2021;2:zqab028 10.1093/function/zqab028 PMC8187230 34124684

[40] Kobak KA, Lawrence MM, Pharaoh G, Borowik AK, Peelor FF, Shipman PD, Griffin TM, Van Remmen H, Miller BF (2021). Determining the contributions of protein synthesis and breakdown to muscle atrophy requires non-steady-state equations. J Cachexia Sarcopenia Muscle.

[41] Miller BF, Drake JC, Naylor B, Price JC, Hamilton KL (2014). The measurement of protein synthesis for assessing proteostasis in studies of slowed aging. Ageing Res Rev.

[42] Zhang S, Zeng X, Ren M, Mao X, Qiao S (2017). Novel metabolic and physiological functions of branched chain amino acids: a review. J Anim Sci Biotechnol.

[43] Yoon MS. The emerging role of branched-chain amino acids in insulin resistance and metabolism. Nutrients. 2016;8 10.3390/nu8070405 PMC4963881 27376324

[44] Kabeya Y, Mizushima N, Ueno T, Yamamoto A, Kirisako T, Noda T, Kominami E, Ohsumi Y, Yoshimori T (2000). LC3, a mammalian homologue of yeast Apg8p, is localized in autophagosome membranes after processing. EMBO J.

[45] Hamilton KL, Miller BF (2017). Mitochondrial proteostasis as a shared characteristic of slowed aging: the importance of considering cell proliferation. J Physiol.

[46] Ahn BH, Kim HS, Song S, Lee IH, Liu J, Vassilopoulos A, Deng CX, Finkel T (2008). A role for the mitochondrial deacetylase Sirt3 in regulating energy homeostasis. Proc Natl Acad Sci U S A.

[47] Martin AS, Abraham DM, Hershberger KA, Bhatt DP, Mao L, Cui H, Liu J, Liu X, Muehlbauer MJ, Grimsrud PA, Locasale JW, Payne RM, Hirschey MD. Nicotinamide mononucleotide requires SIRT3 to improve cardiac function and bioenergetics in a Friedreich’s ataxia cardiomyopathy model. JCI Insight. 2017;2 10.1172/jci.insight.93885 PMC5518566 28724806

[48] Cantrell A, Su H, Zeng H, Chen J-X. Novel regulatory role of SIRT3 on cardiomyocyte mitochondrial frataxin and ferroptosis. The FASEB Journal. 2022;36

[49] Vadvalkar SS, Baily CN, Matsuzaki S, West M, Tesiram YA, Humphries KM (2013). Metabolic inflexibility and protein lysine acetylation in heart mitochondria of a chronic model of type 1 diabetes. Biochem J.

[50] Nacarelli T, Azar A, Altinok O, Orynbayeva Z, Sell C. Rapamycin increases oxidative metabolism and enhances metabolic flexibility in human cardiac fibroblasts. Geroscience. 2018;40(3):243–256 10.1007/s11357-018-0030-2 PMC6060207 29931650

[51] Ingram DK, Roth GS (2021). Glycolytic inhibition: an effective strategy for developing calorie restriction mimetics. Geroscience.

[52] Jing E, O’Neill BT, Rardin MJ, Kleinridders A, Ilkeyeva OR, Ussar S, Bain JR, Lee KY, Verdin EM, Newgard CB, Gibson BW, Kahn CR (2013). Sirt3 regulates metabolic flexibility of skeletal muscle through reversible enzymatic deacetylation. Diabetes.

[53] Trautman ME, Richardson NE, Lamming DW (2022). Protein restriction and branched-chain amino acid restriction promote geroprotective shifts in metabolism. Aging Cell.

[54] Bischof C, Mirtschink P, Yuan T, Wu M, Zhu C, Kaur J, Pham MD, Gonzalez-Gonoggia S, Hammer M, Rogg EM, Sharma R, Bottermann K, Gercken B, Hagag E, Berthonneche C, Sossalla S, Stehr SN, Maxeiner J, Duda MA, Latreille M, Zamboni N, Martelli F, Pedrazzini T, Dimmeler S, Krishnan J. Mitochondrial-cell cycle cross-talk drives endoreplication in heart disease. Sci Transl Med*.* 2021;13:eabi7964 10.1126/scitranslmed.abi7964 34878823

