# Supplementary material for: The loss of cardiac SIRT3 decreases metabolic flexibility and proteostasis in an age-dependent manner
Source: GeroScience. 2022 Dec 3;45(2):983–99. doi: 10.1007/s11357-022-00695-0 (PMC9886736; doi:10.1007/s11357-022-00695-0)
Supplement: Supplementary file 1 — Supplementary file1 (PDF 579 KB) [file 11357_2022_695_MOESM1_ESM.pdf]

Supplementary Figures

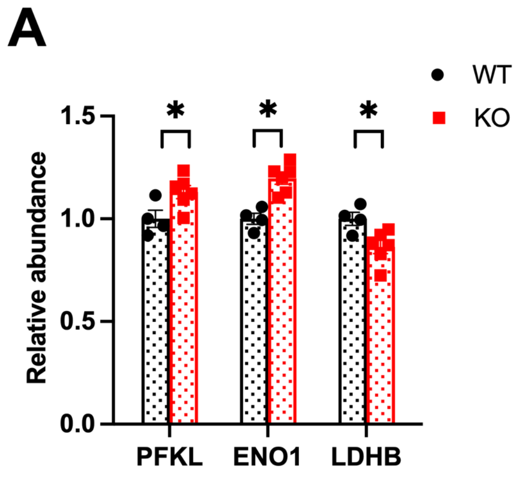

SFigure 1. SIRT3cKO hearts have increased glycolytic enzyme levels and decreased lactate dehydrogenase.
A). Relative protein abundance of PFKL, ENO1, LDHB in heart (n=4-6). Data are presented as mean ± SEM and analyzed using unpaired Student’s t test. *p<0.05.

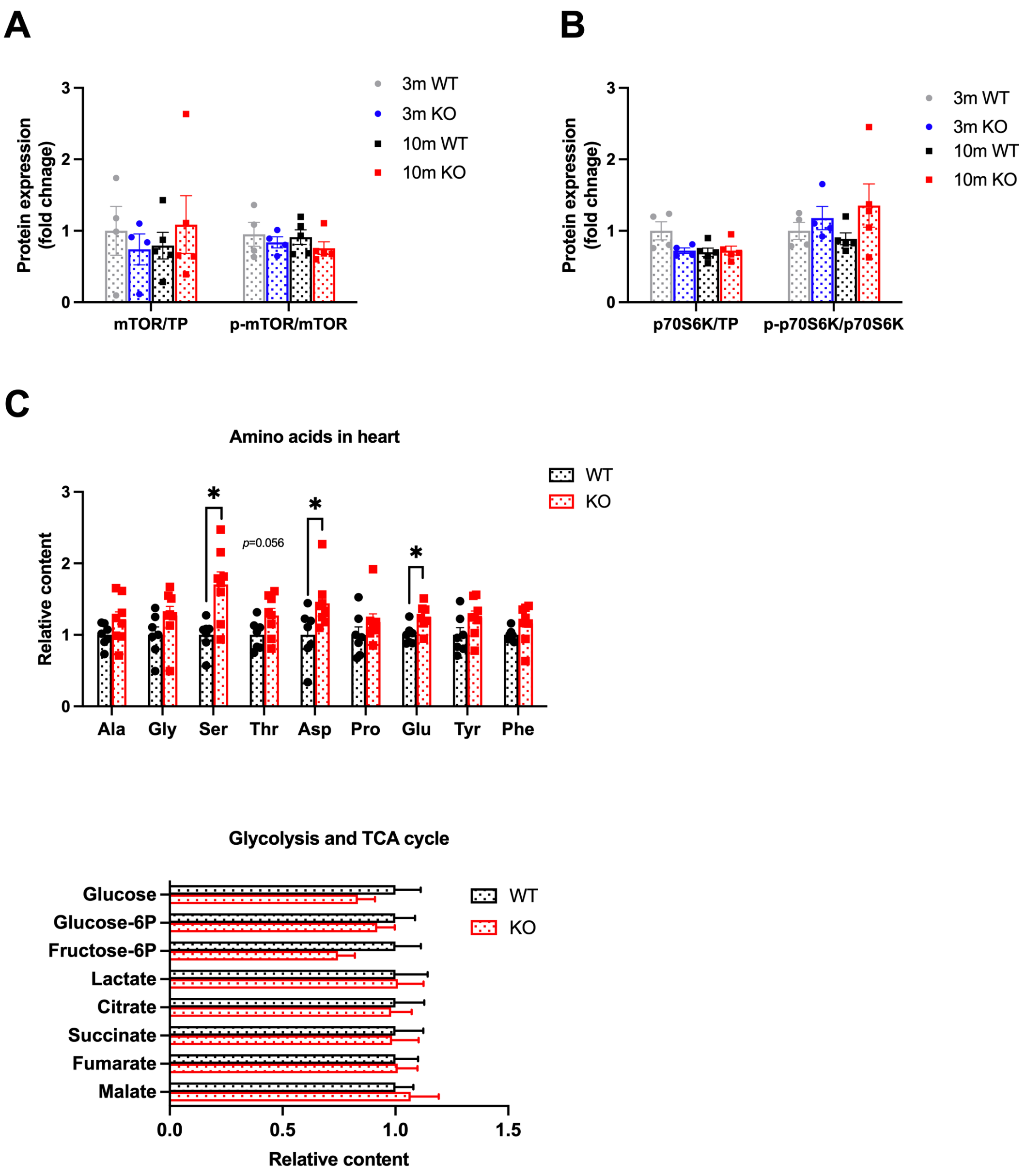

Supplemental Figure 2. mTOR signaling and the levels of central metabolites in SIRT3cKO and WT hearts.

A). Quantitation of phosphorylated and total mTOR ratio, phospho: total mTOR ratio (n=4-5 per group). **B).** Quantitation of total p70-S6K and phospho: total p70-S6K ratio (n=4-5 per group). **C).** Quantification of heart amino acids content, normalized to respective controls *(top)* and quantification of metabolites' content related to glycolysis and TCA cycle, normalized to respective controls*(bottom)* (n=6-8 per group). Data are shown as mean±SEM, and analyzed using 2-way ANOVA followed by Tukey's post hoc test (A-B) and unpaired Student's t test (C). *p<0.05.
